# Muscle in fat trouble: The rise of IMAT in metabolic and musculoskeletal disease

**DOI:** 10.1016/j.molmet.2026.102424

**Published:** 2026-07-16

**Authors:** Vanessa M. Weik, Andreas L. Birkenfeld, Andreas Peter, Cora Weigert, Simon I. Dreher

**Affiliations:** 1 Institute for Clinical Chemistry and Pathobiochemistry, Department for Diagnostic Laboratory Medicine, University Hospital Tübingen, Tübingen, Germany; 2 Institute for Diabetes Research and Metabolic Diseases (IDM) of Helmholtz Munich at the University of Tübingen, Tübingen, Germany; 3 German Center for Diabetes Research (DZD e.V.), Tübingen, Germany; 4 Internal Medicine IV, Department of Diabetology, Endocrinology and Nephrology, University Hospital Tübingen, Tübingen, Germany

**Keywords:** Intermuscular Adipose Tissue (IMAT), Skeletal muscle, Insulin resistance, Type 2 diabetes, Muscle-fat crosstalk, Metabolic disease

## Abstract

Intermuscular adipose tissue (IMAT) is increasingly recognized as a contributor to insulin resistance and metabolic dysfunction in type 2 diabetes (T2D). Accumulation of IMAT was found to correlate with impaired skeletal muscle insulin sensitivity, as well as a generally reduced muscle strength and physical performance in humans. Beyond serving as an energy depot at physiological levels, increased IMAT is thought to actively impair muscle metabolism through secretion of adipokines, cytokines and lipid intermediates that create an inflammatory environment and modulate insulin signaling pathways. This review discusses current evidence on the pathophysiological role of IMAT based on clinical studies, including interventions, and current mechanistic insight from biopsied human IMAT. We further explore traditional and emerging methods to investigate IMAT that could expand mechanistic understanding of IMAT-muscle-crosstalk, highlighting their strengths and limitations*.* Human *in vitro* co-culture-models are valuable future tools for dissecting cellular and molecular responses. Despite growing interest in IMAT as a potential key player in metabolic disease, uncertainty remains about its origin, regulation and functionality. We suggest further research to integrate and intertwine traditional imaging techniques, multi-omics characterization of IMAT biopsies and advanced, physiologically relevant human *in vitro* systems to close these knowledge gaps to develop therapeutic strategies targeting metabolic disease.

## 1 The marbled nature: IMAT, not just any other fat?

**Figure 1:**
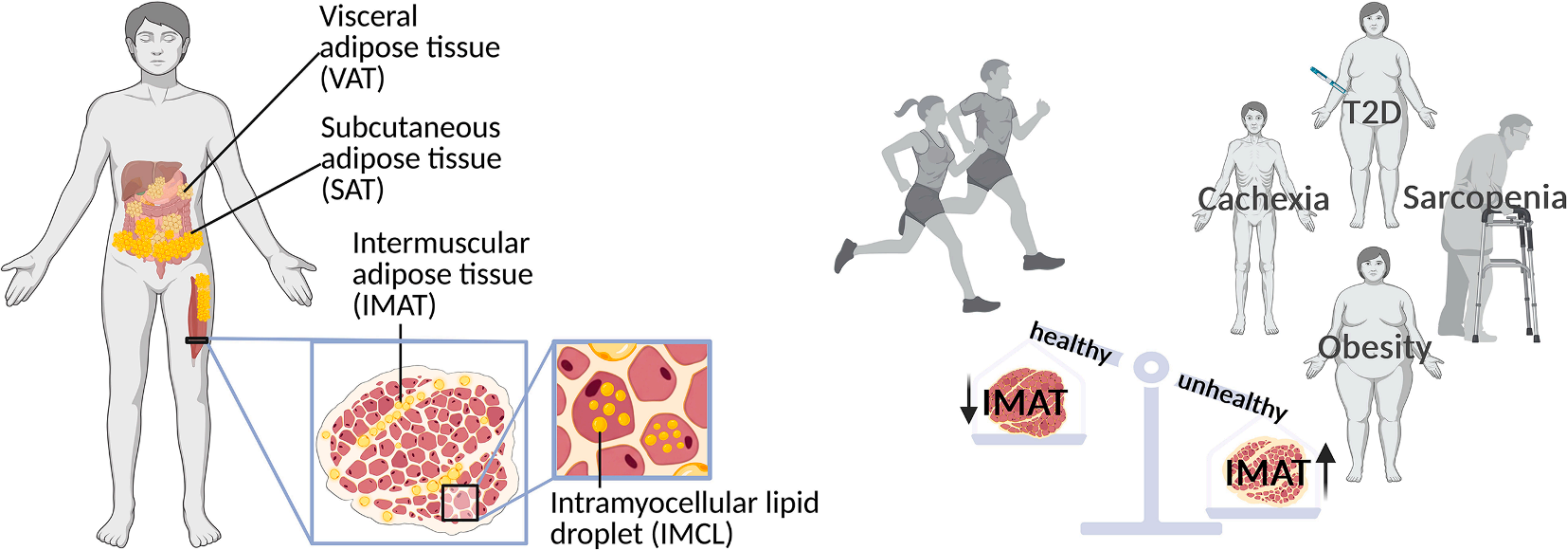
Overview over the human bodies' main adipose tissue depots and adipocyte/lipid storage in skeletal muscle (left). IMAT is generally regarded as a nearby energy depot for healthy skeletal muscle cells. Elevated levels of IMAT are associated with metabolic disease and muscle wasting (right). Figure created with biorender.com.

Skeletal muscle is the largest organ in the human body by mass, making up approximately 40 % of total body weight [1]. Furthermore, it accounts for the majority of insulin-stimulated glucose uptake and is also highly involved in lipid metabolism [2,3]. Lipids can accumulate in muscle through different distinct depots: as intramyocellular lipids (IMCL), stored as triglyceride-containing droplets within muscle cells and as intermuscular adipose tissue (IMAT), located between muscle fiber bundles beneath the deep muscle fascia (Figure 1) [4,5]. The muscular fat depots have been positively correlated with an increased body fat content in obesity, just like subcutaneous and visceral adipose tissue (SAT and VAT, respectively) [6]. IMCL, IMAT, VAT and SAT have additionally been linked to insulin resistance and type 2 diabetes mellitus (T2D) [[5], [6], [7], [8], [9], [10]]. Curiously, while IMCL levels are elevated in sedentary and obese individuals, endurance trained athletes have been documented to possess high levels of IMCL too [8]. Recent findings unveiled that athletes exhibit higher intramyocellular lipid saturation and turnover, which supports mitochondrial oxidation, while IMCL are unsaturated in T2D but can turn increasingly saturated through endurance training [11,12]. This finding suggests that IMCL is not only possibly involved in insulin resistance or type 2 diabetes but acts as an essential fuel depot for the oxidative metabolism of muscle [11,13,14]. In contrast, this athlete's paradox seemingly does not apply to IMAT. Instead, in comparison to IMCL, IMAT is independently linked to insulin resistance, impaired glucose tolerance and reduced muscle strength, slower gait speed and poorer exercise performance, highlighting it as a better marker of metabolic dysfunction and muscle quality [7,15]. It is a well-vascularized tissue, predominantly composed of clusters of white adipocytes beneath the deep muscle fascia with interspersions of preadipocytes, fibroblasts, immune cells and endothelial cells [[16], [17], [18], [19]]. The adipogenic expression of perilipins and adipocyte markers such as fatty acid binding protein 4 (FABP4) and peroxisome proliferator-activated receptor γ (PPARγ) characterize IMAT-adipocytes as mature [20]. Oil Red O staining reveals a morphology and lipid droplet structure similar to that of white adipose cells [21]. This review will focus on IMAT as a key ectopic fat depot that links skeletal muscle metabolism to insulin resistance and diabetes development and as a potential target for metabolic disease treatment.

## 2 Fat where it should not be?

IMAT distinguishes itself from other lipid depots due to its unique localization beneath the muscle fascia. The close proximity to skeletal muscle cells reflects its physiological function. IMAT is thought to contribute to local lipid availability of muscle cells by acting as a nearby supplier of fatty acids and therefore an energy source [9]. As limited information on IMAT is available, it is generally presumed to function analogously to other regional adipose tissue depots by secreting adipokines, cytokines and free fatty acids (FFAs) that participate in the regulation of muscle metabolism and function [9,22]. Beyond metabolic signaling, IMAT, embedded within muscle, may exert structural and mechanical cues by influencing muscle fiber spacing, ECM properties and muscle vascularization [23]. In this way, even though it represents a small fraction of total adipose tissue, IMAT can impact muscle energy utilization and tissue homeostasis.

### 2.1 IMAT and the breaking point: when fat hijacks muscle function

Although IMAT displays physiological functions in muscle metabolism, its expansion to a threshold of 6.8–7.5% of skeletal muscle area has been associated with metabolic and functional impairment [24,25]. This correlation has been found in multiple pathological and age-related conditions, including T2D, sarcopenia, cachexia and primary myopathies, during which muscle volume has been observed to be gradually replaced by IMAT (Figure 1) [15,[26], [27], [28], [29]]. Current efforts to combat muscle wasting focus on the preservation of muscle mass and neglect IMAT infiltration due to limited knowledge about IMAT and how it forms. During aging and sarcopenia, IMAT volume increases even without the onset of obesity or major changes in body mass [30]. This suggests that the expansion of IMAT goes hand in hand with the disuse and the resulting neuromuscular remodeling, rather than just adiposity [28,31]. Imaging studies have consistently found an association between increased muscle fat infiltration in sarcopenic older adults and a decreased muscle strength and gait speed [32]. As elevated IMAT is associated with lower muscle performance, a link between IMAT accumulation and peripheral neuropathy in patients with diabetes is suggested [33]. IMAT intervention might alleviate peripheral neuropathy in diabetes. Inversely, peripheral neuropathy may further contribute to IMAT formation, as the subsequent reduced motor activity promotes muscle atrophy and therefore IMAT expansion. Generally, rather than muscle mass, muscle strength has been increasingly linked with muscle quality which is considered to be negatively associated with IMAT content [[34], [35], [36]]. In the context of T2D, IMAT accumulation has been found to correlate strongly with insulin resistance and impaired glucose tolerance, regardless of total body mass and content of other fat depots, like VAT [7,37,38]. Long-term cohort studies also demonstrate that subjects with higher baseline IMAT, whether due to genetic or lifestyle factors, not only display a higher degree of insulin resistance but also experience a faster decline in physical performance and mobility [33,39,40].

Pathological IMAT infiltration is not only restricted to aging and metabolic diseases. In cancer cachexia, muscular dystrophies and myopathies, adipose tissue progressively replaces functional muscle tissue, bringing about impaired muscle function and regeneration [27,29]. In these conditions, IMAT is a marker for muscle loss and is considered part of the disease process.

Some evidence suggests sex-specific differences in IMAT deposition. Interestingly, these differences do not mirror the well-established sexual dimorphism in visceral adiposity, as males generally accumulate greater amounts of VAT [41] whereas females are considered to have higher levels of muscle fat infiltration than males, in particular when IMAT mass is related to total muscle mass [[42], [43], [44], [45]]. Females have a greater triglyceride extraction from very low density lipoprotein (VLDL) in skeletal muscle, which can drive the higher lipid content in skeletal muscle stored as IMAT, but also IMCL [[46], [47], [48]]. On the other hand, lipolysis of IMCL was reported to be higher in females, well in line with higher fat oxidation in the female skeletal muscle during exercise [[49], [50], [51]]. This increased turnover rate of lipids stored in the skeletal muscle fat depots potentially explains why the higher IMAT ratio in females is not associated with reduced insulin sensitivity. Pre-menopausal females show even better insulin sensitivity than males at similar fitness and display greater insulin-dependent glucose uptake in skeletal muscle [52]. Hormones, especially estrogen, can contribute to this apparent paradox [52]. Rodent models suggest that estrogen can enhance insulin sensitivity [53]. This positive estrogen-effect might be transferable to humans since post-menopausal, low estrogen women show an increase in IMAT and also VAT deposition and declining insulin sensitivity, whereas estrogen replacement therapy improves glycemic control [[54], [55], [56]]. While sex-specific differences in the metabolic consequences of IMAT seem apparent, less is known regarding potential sex differences in the relationship between IMAT and muscle function. Whether females exhibit reduced IMAT-associated impairments in physical performance remains mostly unexplored.

Collectively, these findings indicate that IMAT accumulation is a critical point linked to muscle health. It is associated with reduced muscle strength, slower gait speed and impaired mobility, particularly in older individuals and those with metabolic disorders. Its association with insulin resistance positions IMAT as a potential key mediator in metabolic disease.

### 2.2 In search of causality: is IMAT a marker or a mediator?

Although numerous studies have documented a strong correlation between IMAT accumulation and insulin resistance, T2D, reduced muscle strength and mobility, it remains unclear whether IMAT acts as a causal mediator or is merely a biomarker. Cross-sectional human studies frequently display a correlation between increased IMAT content, unrelated to total fat mass, lower insulin sensitivity and higher inflammatory marker expression [7] [57]. However, as much of the evidence in humans is associative, these correlative observations cannot determine causality. The results of interventional studies can provide stronger leads. Weight loss interventions lead to enhanced insulin sensitivity accompanied by a reduction of IMAT volume and improved glucose uptake [[58], [59], [60]]. Additionally, weight loss through dietary restrictions alone has shown lesser effects than weight loss accompanied by exercise, highlighting a connection between IMAT content and regular physical activity [59,60]. Reciprocally, physical inactivity rapidly increases IMAT and coincides with decreased local insulin sensitivity and lower oxidative capacity of muscle [39]. These findings together suggest a functional coupling between changes in IMAT mass and muscle metabolism. Though most clinical data as of now measure composite muscle fat using MRI/computed tomography (CT) rather than isolated IMAT, they still provide suggestions that changes in IMAT content are dynamically coupled to muscle metabolic health. An *in vitro* study performed by Sachs et al., in which primary human myotubes were cultivated in IMAT-conditioned media, obtained by cultivating IMAT from human biopsies, resulted in a decrease of insulin sensitivity in those myotubes. To date, this provides the strongest direct hint at IMAT influencing muscle metabolism through its secretome [15]. While IMAT and other fat depots are closely correlated in humans, emerging evidence further suggests unique properties of IMAT. A recent study identified depot-specific secretory characteristics. The IMAT-derived secretome has been found to differ from VAT despite both affecting skeletal muscle insulin sensitivity [15,22]. These findings suggest that IMAT's biological effects can be considered independently from VAT. Nevertheless, causality remains difficult to establish in humans due to the invasive nature of tissue sampling and application of imaging techniques with limited resolution to isolate IMAT from other lipid depots in muscle. There is little longitudinal data on changes in IMAT and other fat depots, and it is unclear whether alterations in IMAT precede changes in insulin sensitivity or simply result from metabolic adaptations. While causal links between IMAT and metabolic dysfunction are emerging, comparable mechanistic evidence for direct effects of IMAT on muscle strength and muscle performance is more limited. Currently, functional impairments of skeletal muscle associated with IMAT are often suggested to stem from changes in muscle composition and spatial effects [23].

## 3 Saboteur signals: molecular crosstalk between IMAT and muscle

**Figure 2:**
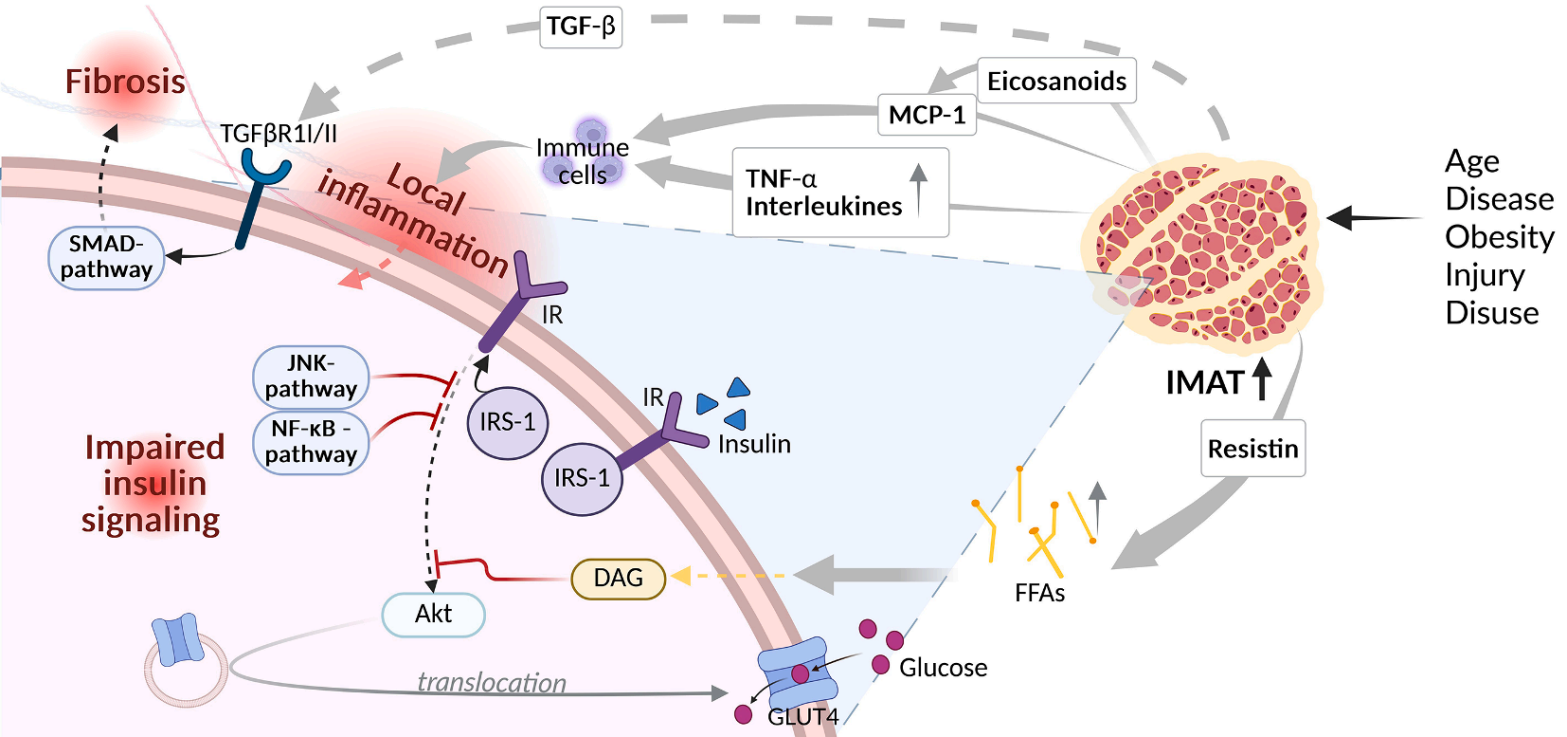
Schematic overview over signaling between IMAT and muscle cells. Metabolic disease and muscle wasting causes an increase in IMAT volume in skeletal muscle. IMAT secretome was identified to contain high levels of MCP-1 which recruits immune cells that cause local inflammation in the muscle environment. This inflammatory reaction might impair insulin signaling and insulin-mediated glucose uptake. IMAT-secreted resistin causes an increase in adipocyte-produced FFAs that stunt insulin-mediated glucose uptake via inhibition of the insulin pathway in skeletal muscle cells. Further potentially detrimental cytokines released by IMAT contain factors like TNF-α and TGF-β. Figure created with biorender.com.

Recognizing IMAT as being more than a metabolic marker shifts the focus towards the mechanisms by which IMAT could cause metabolic disruption. As *in vitro* experiments using IMAT-conditioned media, obtained by cultivating IMAT from human biopsies, resulted in a decrease of insulin sensitivity in human myotubes [15], IMAT likely asserts influence on muscle fibers through paracrine signaling. Proteome analysis using the Olink platform of the secretome of human adipose tissue depots, isolated from biopsies of obese individuals, has shown that IMAT secretes higher levels of cytokines and chemokines, such as TNF-α, various interleukins and monocyte chemotactic protein 1 (MCP-1), as well as adipokines including resistin and leptin than other adipose tissue depots [22] (Figure 2).

Chronic low-grade inflammation, triggered and upheld by these IMAT cytokines, has been linked to obesity, insulin resistance and T2D. It disrupts insulin action by interfering with the insulin signaling cascade at the level of the insulin receptor and its substrates, thereby preventing phosphatidylinositol 3-kinase (PI3K)/Protein Kinase B (AKT)-activation, GLUT4 translocation and GLUT4-mediated glucose uptake [61,62].

MCP-1 is secreted in particularly high levels in IMAT [22,57]. Its primary role is the recruitment of monocytes/macrophages to inflammation sites in immune responses, creating a pro-inflammatory environment [63]. MCP-1 knockout studies performed in mice provide evidence that MCP-1, even in small amounts, contributes to insulin resistance [64,65]. This effect might stem from the amplification of local inflammation, further disrupting insulin-stimulated glucose uptake. The adipokine resistin is also secreted in large quantities by IMAT [22]. The mechanism of interaction between resistin and muscle cells has not yet been clarified, though, resistin has been described to alter phosphorylation of IRS-1 and therefore GLUT4 translocation and glucose uptake in rat myotubes [66]. These findings have not been replicated in human skeletal muscle. Instead, an increased level of resistin has been correlated with promotion of lipolysis *in vitro*, possibly hinting at an increased release of circulating free fatty acids (FFAs) by IMAT [67,68]. FFAs have long since been revealed to decrease glycolysis through metabolic and signaling pathways. FFAs are metabolized into lipid intermediates, such as diacylglycerol (DAG), which inhibit insulin-induced phosphorylation of AKT, leading to impaired PI3K/AKT-signaling and reduced GLUT4 translocation [69]. Additionally, the IMAT secretome displays elevated levels of eicosanoids like 12-hydroxyeicosatetraenoic acid (12-HETE) [22]. These eicosanoids promote inflammatory responses that can trigger MCP-1 and therefore can be related to insulin resistance [70,71].

This IMAT-induced inflammatory environment not only affects insulin sensitivity, but also muscle regeneration and growth. A co-culture of SAT/VAT-derived adipocytes and muscle cells has revealed elevated levels of cytokines (IL-6) and chemokines, creating a pro-inflammatory environment that causes muscle atrophy and a decrease in expression of contractile muscle proteins [72]. As VAT and IMAT share a similar secretome, IMAT is very likely to induce muscle atrophy too [22,72]. Moreover, the correlation between high levels of IMAT and impaired muscle function suggests that growth factor signaling might be altered in IMAT-rich muscle. Transforming growth factor beta (TGF-β) is a prime candidate in this scenario, since it causes excess deposition of ECM proteins and an increase in myofibroblast differentiation which reduces muscle elasticity and impairment of muscle satellite cell (SC) activation [[73], [74], [75]]. While there is no direct evidence that documents human IMAT as a source of excessive TGF-β, adipose tissue in obese mice and humans has been shown to express it [[76], [77], [78]]. Combining the possibility of IMAT as a source of TGF-β signaling and its biological function, IMAT-secreted TGF-β might contribute to loss of muscle function by muscle stiffening in humans as well, corresponding to the observations in the aforementioned IMAT human cohort studies [28]. The combination of impaired muscle protein expression caused by IMAT-derived cytokines and the TGF-β-driven fibrosis cannot only impair muscle regeneration but might also create an environment that further favors muscle fat infiltration in metabolic disease.

Summarized, from a pathological perspective, IMAT is currently understood to secrete comparatively high amounts of cytokines and chemokines that trigger a chronic local inflammatory environment which disrupts insulin signaling in neighboring muscle cells and muscle growth.

## 4 Clinical outlook: can we target IMAT?

While *in vitro* studies using biopsies have provided further insight into cellular mechanisms that drive IMAT formation and IMAT's potential causal role in metabolic dysfunction, the challenge of translating this information into clinical benefit stands.

### 4.1 The pharmacological route

Metabolic pharmacotherapy has shown great potential in diabetes treatment and reduction of visceral and total fat for obesity treatment. Among these pharmaceutical actives Metformin, a biguanide of herbal origin, is the first-line drug for treatment of diabetes and exerts its blood glucose level-lowering effect through a decrease in hepatic glucose production and an increase in cellular glucose uptake [[79], [80], [81]]. While there is no research on the influence of metformin on IMAT, findings of correlative and murine studies suggest that metformin might suppress adipogenic differentiation which could affect IMAT deposition, though this remains to be validated [82].

Current attention in T2D and obesity treatment is directed towards incretin-based therapies. These mono-und dual-incretin receptor agonists have a strong SAT and VAT lowering potential and it is plausible that these effects extend to IMAT [83,84]. First MRI analyses report a noticeable reduction of IMAT content after 52 weeks of treatment of obese individuals with dual GIP-/GLP-1 agonists [85]. Another study using a 40-week mono-GLP-1-receptor agonist-treatment has observed a reduction of muscle fat infiltration in human thigh muscle of obese individuals [86]. In contrast, a study using pre- and post-semaglutide treatment CT-scans of obese patients in combination with an AI body composition tool has found the decrease of IMAT through semaglutide to be minimal compared to the patients’ loss of VAT and SAT [87]. Although incretin-based therapies have been shown to consistently improve metabolic outcomes and potentially reduce IMAT, their impact on muscle strength and physical performance is less well understood. Furthermore, incretin-based therapies lead to a significant reduction of muscle mass with recent data suggesting a loss of around 25% of total weight loss being lean mass [[88], [89], [90]]. This weight-loss-induced reduction in muscle mass could result in adverse effects as fat re-accumulation often occurs more rapidly than muscle hypertrophy resulting in a less favorable IMAT-to-muscle ratio [91]. This topic is currently highly discussed with further research being underway, possibly clarifying the effect of GLP-1 agonist discontinuation on IMAT accumulation. Future trials combining GLP-1 agonists with specified training regimens or anabolic co-treatments with e.g. myostatin inhibitors will be crucial to determine whether IMAT reduction can be sustained or reversed post therapy without muscle loss. No pharmacological treatment to date is independently validated to specifically target IMAT in humans.

### 4.2 The lifestyle route

Lifestyle intervention studies associated with weight loss have also shown a reduction in IMAT in most cases. Both caloric restriction [59,60,[92], [93], [94], [95]] and weight loss through combined diet and exercise [59,60,96] have been shown to reduce IMAT content. Weight loss appears to be the main determinant of IMAT reduction, but interventions that combine weight loss with regular physical activity frequently demonstrate favorable effects on IMAT and metabolic health outcome such as improved insulin sensitivity and glucose uptake [59,60]. Furthermore, long-term exercise interventions have been associated with the prevention of IMAT accumulation along improvements in metabolic parameters [97,98]. A meta-analysis across different cohort studies has found that weight loss in combination with aerobic exercise alone, or combined with resistance training reduces IMAT in individuals aged 18–65 [99]. Conversely, physical inactivity has been shown to increase IMAT accumulation which is accompanied by reduced insulin sensitivity, loss of muscle mass and oxidative capacity [39]. This indicates that the mechanical loading of muscle strongly influences IMAT accumulation.

**Figure 3:**
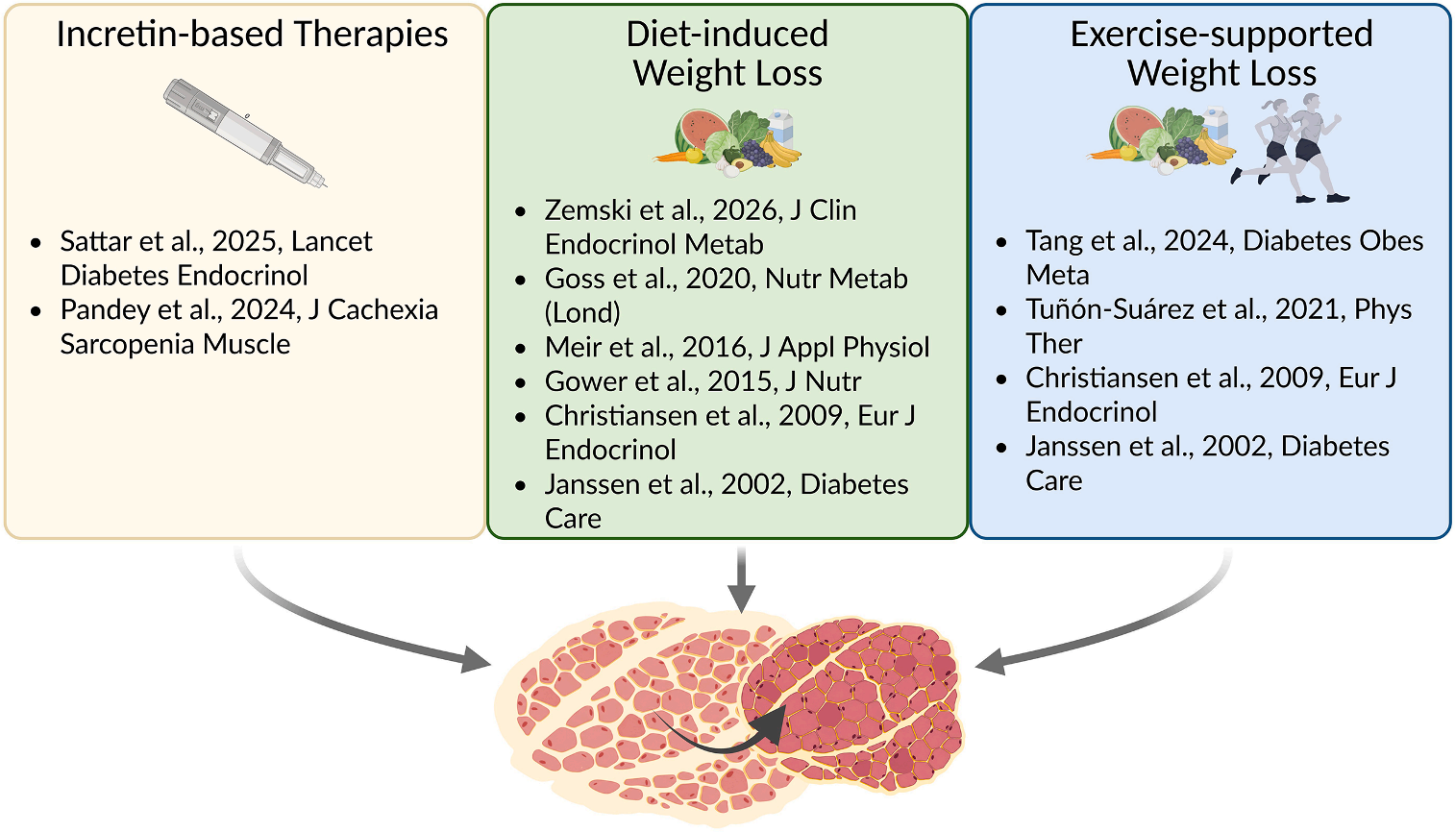
Categorization of intervention studies that reportedly targeted and reduced human IMAT. Figure created with biorender.com.

Collectively these lifestyle intervention studies suggest that reductions in IMAT are consistently achieved through weight loss (Figure 3). While the independent contribution of exercise to reducing IMAT without weight loss is considered minimal [59,60,92,95,97], exercise remains highly relevant as a therapeutic strategy as it improves insulin sensitivity and most importantly preserves muscle function which may prevent the progressive accumulation of IMAT over time.

Together, emerging evidence supports IMAT as a modifiable therapeutic target, rather than a biomarker. The next steps in IMAT research should provide more insight into IMAT reduction and discuss the question of IMAT as a pathological or adaptive tissue to a greater extent. Further research is needed into additional strategies to preserve muscle mass while reducing muscle-fat infiltration. The most promising outcomes in metabolic health are likely to be achieved through combinations of exercise- and pharmacologically-supported weight loss treatment.

## 5 Where does IMAT come from?

**Figure 4:**
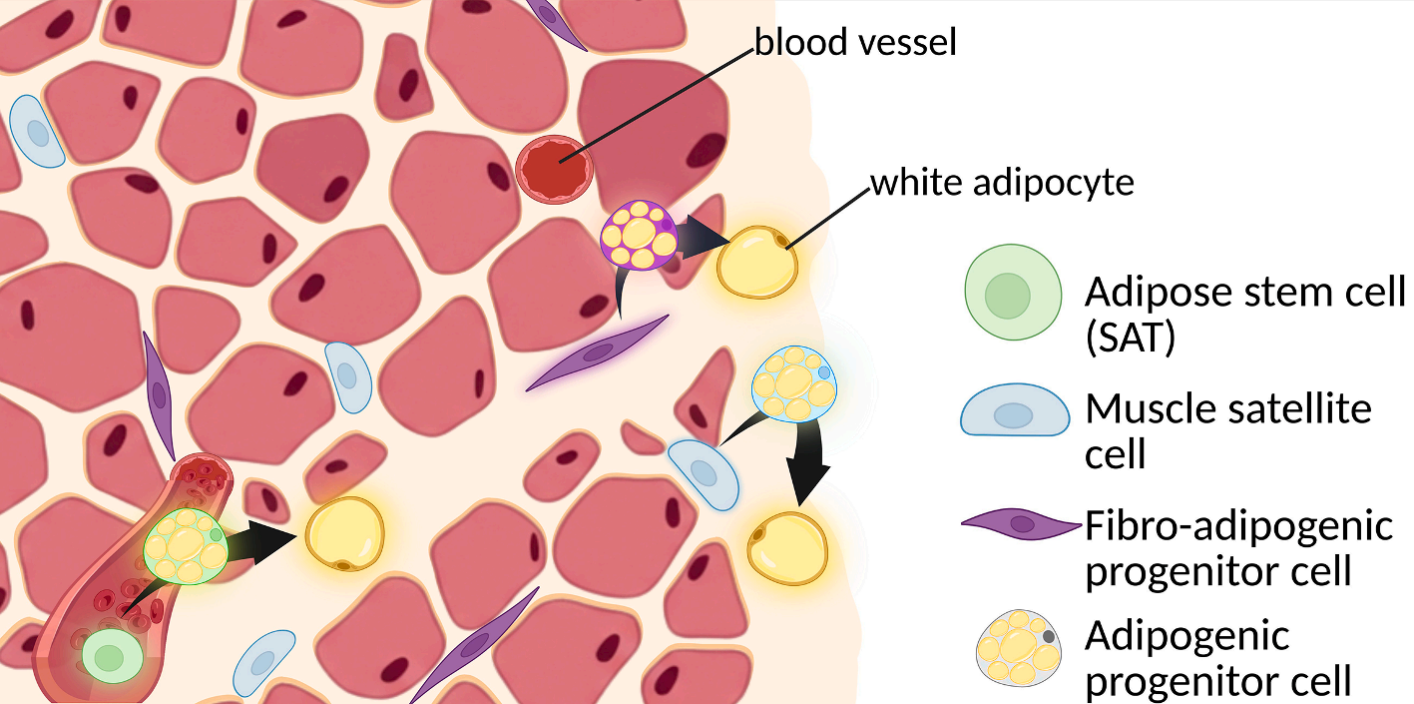
Skeletal muscle cross section with myotubes (red) surrounded by IMAT (light yellow). Muscle SC (light blue) and FAPs (purple) are situated interstitially and are indicated to differentiate into white adipocytes via an adipogenic progenitor cell corresponding to the color code. SAT-derived ASCs enter the muscle through blood vessels and differentiate into white adipocytes that contribute to IMAT. Figure created with biorender.com.

To understand the cellular origin of IMAT is of high relevance for developing strategies with the aim to reduce IMAT formation or modulate its function. Early studies suggested that adipocytes within skeletal muscle might descend from transdifferentiating muscle SCs [100,101]. This assumption was supported by the observation that *in vitro* human muscle SCs develop lipid droplets when treated with adipogenic differentiation factors [101]. Circulating adipose stem cells (ASCs), originating from SAT, were identified as a contributor to IMAT formation in response to muscle injury in mice [102]. Though this has not been translated into a human system, an *in vitro* experiment using primary human cells has shown that human ASCs migrate towards blood serum containing signals of muscle exhaustion/injury [103]. This indicates that SAT-derived ASCs might also follow a muscle injury chemotaxis in humans and contribute to IMAT formation. More recent findings suggest fibro-adipogenic progenitors (FAPs) as the origin of IMAT [104]. In healthy conditions, FAPs support muscle regeneration by secreting trophic factors, such as insulin-like growth factor 1 (IGF-1) and IL-6 [105]. Under pathological stress, they were shown to differentiate into adipocytes, possibly accounting for IMAT, or into myofibroblasts, leading to the fibrosis of muscles [105]. Interestingly, FAPs share a similar adipogenic capacity and differentiation cues with adipogenic progenitors derived from VAT and SAT, with the deciding factor for the adipogenic differentiation being the transcription factor PPARγ [21]. In general, during adipogenesis, external cues, like nutrients or insulin, activate early adipogenic transcription factors CCAAT-enhancer-binding proteins beta and delta (C/EBPβ and C/EBPδ) [106]. These activate PPARγ which induces adipogenic maturation through activation of genes for lipid uptake, lipid droplet formation and adiponectin, which is involved in the regulation of insulin sensitivity [107,108]. *In vitro* experiments using primary human FAPs, treated with adipogenic differentiation factors, have resulted in mature, insulin-resistant white adipocytes, suggesting a common adipogenic differentiation process [21]. The different contributions of SC, ASC and FAPs to formation of IMAT in healthy conditions in humans and how this process is altered in metabolic disease, muscle injury and aging needs to be elucidated (Figure 4).

## 6 How can we study IMAT?

Having outlined the proposed cellular origins of human IMAT and the significance of IMAT for clinical research, the question arises: how can we investigate the amount, function and quality of IMAT and thereby establish it as a clinical target?

### 6.1 Let's take a picture

**Figure 5:**
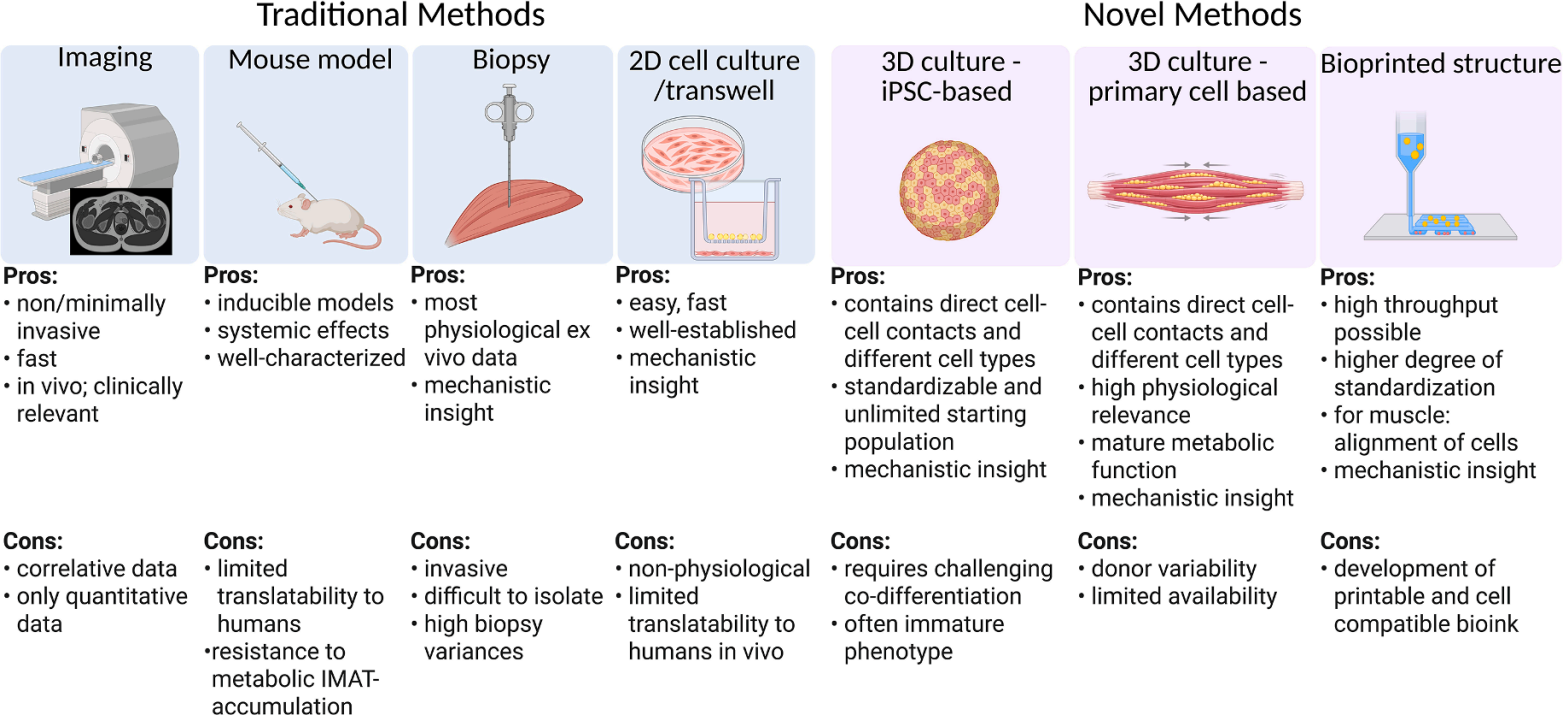
Comparison of traditional and novel methods to study IMAT and their pros and cons. Figure created with biorender.com, 3D culture – primary cell based image generated by DALL·E 3.

The first studies to describe IMAT as an independent fat depot were conducted using imaging techniques. These techniques unveiled correlations between IMAT and metabolic diseases in the year 2000 [7]. While initial descriptions of IMAT have already surfaced at the start of the 21st century, many early magnetic resonance-based studies did not use the term IMAT yet, but instead describe extramyocellular lipid (EMCL) depots within the muscle compartment which are included in IMAT [[109], [110], [111]]. Presently, measurement of IMAT is most commonly executed via magnetic resonance imaging (MRI), computed tomography (CT) and as of lately via ultrasound [4,7,112]. MRI permits the direct measurement of IMAT without ionizing radiation and differentiates fat and muscle based on their proton relaxation properties, measuring their nuclear magnetic resonance [113]. CT presents a faster, more indirect method by measuring tissue density. In the context of IMAT measurement, muscle is imaged as high-density and fat as low-density tissue [114]. Among the imaging methods, MRI is considered the reference standard, as it provides superior soft tissue imaging [4]. A newly emerging IMAT imaging method utilizes ultrasound with which changes in muscle echogenicity can be measured as an indicator for IMAT content. However, this method is limited by the inability to distinguish between IMAT and IMCL and the strong dependence on the ultrasound-operator [112]. Ultimately, IMAT imaging only provides correlative data between IMAT content and metabolic dysfunction (Figure 5). Mechanistic insight is only gained through IMAT tissue sampling that allows molecular profiling of cellular components and analysis of its secretome. However, as it is challenging to obtain a biopsy of a small, localized area of tissue, such as IMAT, studies using human IMAT biopsies are scarce. Biopsied IMAT has only been used in very few studies with the experiments by Sachs et al., in 2019, discussed in chapter 3, being the first case of a direct human IMAT sampling study with continued use of those samples for further research [15,115]. The same research group then performed a second IMAT biopsy for secretome analysis [22]. IMAT's transcriptome has been described to differ from other fat depots in two works using biopsied IMAT [115,116]. Another study used IMAT samples to study IMAT-derived macrophages [117]. In most cases, the biopsies were taken during surgical procedures, underlining the difficulty of obtaining human IMAT.

### 6.2 Of mice and men

Historically, animal models have been instrumental in studying basic mechanisms and disease models in ways that are not possible in humans. However, currently known mouse strains are resistant to IMAT formation during high fat diet, rarely develop IMAT in age or metabolic stress and mostly produce IMAT through muscle injury models, such as the injection of glycerol or tendon rupture [118,119]. Using these approaches, murine models have also contributed to our understanding of the cellular origins and functional consequences of IMAT formation, including direct impairments in muscle strength [36]. Furthermore, murine studies show a phenotypic plasticity of IMAT progenitors, specifically an indication of browning, which suggests IMAT to be a modifiable fat depot and a potential therapeutic target [16,120]. Although these observations could provide valuable preclinical information, the use of murine models has limitations when it comes to studying the impact of IMAT accumulation in metabolic diseases. (Figure 5).

### 6.3 Bench-top encounter: *In vitro* systems for IMAT-muscle crosstalk

To attain better mechanistic insight into the effects of IMAT on skeletal muscle, without relying on IMAT-resistant mice, *in vitro* studies are indispensable. Consequently, co-culture systems of fat derived from ASCs or mature adipocytes and muscle have been established to further improve the understanding of the metabolic crosstalk between fat and muscle with the objective of gaining insight into pathways that can be intervened for medical applications, such as the treatment of obesity or T2D [121,122] (Figure 5). Early *in vitro* studies using transwells or cell-conditioned media demonstrated that the secretome of human IMAT- and 3T3-L1-adipocytes can blunt insulin-stimulated glucose uptake and impair mitochondrial function in respective myotubes, as well as disturb muscle cell growth [15,116,123]. These approaches fail to include the spatial organization, continuous signaling and direct cell contacts of IMAT and muscle *in vivo.* A more advanced model, specifically for the research of muscle-fat interactions which utilizes self-assembled 3D structures, has been developed by Shahin-Shamsabadi et al. [124]. The model is comprised of partially differentiated murine C2C12 and 3T3-L1 cells that are encased in a collagen type I hydrogel, either in direct contact by using a mixture of both cell lines in the bioink or in indirect contact by depositing overlapping hydrogel constructs of a singular cell type. Using this model, it was shown that direct contact results in a reduced differentiation of muscle cells and a reduced lipolysis of fat cells when treated with anti-obesity drugs in contrast to indirect contact [124]. This stresses the importance of a direct physical-contact IMAT model, as these observations cannot be obtained using indirect contact. While this model includes signaling by cell-cell-connection, it does not include the functional aspects of muscle. Even though there has been progress in integrating adipose tissue or adipose microfibers with engineered muscle constructs [125], to our knowledge, no research to date has demonstrated a fully functional human 3D muscle-IMAT construct. Most co-culture work remains in 2D indirect cultures. This represents a gap for modeling IMAT for clinically relevant research.

### 6.4 A 3D model says more than a thousand cells

#### 6.4.1 Muscle culture: *In vitro* muscle models with the potential to study IMAT crosstalk

To fill this gap and generate a fully functional 3D IMAT model, the obvious approach would be the combination of established 3D models of each component, fat and muscle. While both adipocytes and myotubes have been successfully cultured *in vitro*, with established cell lines being widely available, the cultivation of myotubes faces special challenges [126]. As with contracting myofibers *in vivo*, the secretome and expression profile of muscle cells changes in response to contraction *in vitro*, which makes contractility an important quality measure of physiologically relevant muscle models [127]. The most common cultivation method is a 2D culture system where immortalized or primary murine/human myoblasts are differentiated into multinucleated myotubes [128,129]. These cultures are easy to achieve and maintain and have historically provided valuable insights into molecular mechanisms in cells [130]. However, 2D muscle cultures exhibit functional limitations and do not reproduce the 3D organization, ECM-composition or mechanical loading present *in vivo* that are considered critical for myofiber maturation. *In vitro* analysis of 2D grown human myotubes describe immature structural organization, sarcomere formation and gene expression [126]. These limitations have accelerated the transition toward 3D culture systems that better recapitulate *in vivo* architecture, mechanical cues and ECM composition [131]. Native skeletal muscle comprises structural ECM proteins such as collagen, elastin and glycoproteins and includes vascular, neural and immune components, which remain difficult to integrate *in vitro* [132,133]. Muscle organoids partially address this complexity through self-assembly and differentiation into multiple cell types (Figure 5), though no fat-muscle organoid has been described to date. Pluripotent stem cell–derived spheroids can be differentiated into neuromuscular organoids containing spinal cord neurons and muscle cells with functional connections [134,135]. However, organoids do not fully reproduce muscle fiber organization, contractile function or tension-driven maturation. Tissue-engineered muscle constructs overcome these limitations by combining myogenic cells with ECM-mimicking biomaterial scaffolds under uniaxial tension [136] (Figure 5). Embedded precursor cells differentiate into aligned, contractile myofibers responsive to electrical stimulation (EPS), commonly using collagen I, fibrin or Matrigel hydrogels [131,137,138]. The resulting “myobundles” exhibit multinucleation, sarcomere maturation and adult myosin isoforms, enabling functional analyses such as force generation, fatigue resistance and metabolic performance [129,131,139]. Despite these advantages, myobundles remain limited by their size and their exclusion of other cell types [140]. Another common challenge of 3D cultures is their variability in cell density, fiber alignment, scaffold composition, stiffness and nutrient diffusion. This variability complicates experimental reproduction [141]. Additionally, although manual micromolding is methodically simple and cost-effective, it demonstrates low scalability. Bioprinting technology addresses these issues by enabling machinated fine-tuning and precision in large scales [142] (Figure 5). Extrusion-based bioprinting, one of the most established techniques [143], aligns bioink-encapsulated cells along the printing axis through nozzle-induced shear stress, promoting myofiber formation and making it suitable for myotube generation [144]. Current limitations mainly relate to bioink biocompatibility. Highly printable hydrogels are dense and poorly porous, restricting cell migration, myoblast fusion and nutrient diffusion. Blending printable and cell-compatible materials, such as alginate–gelatin composites, can improve these properties [145,146]. Functional bioprinted myotubes with contractile capacity comparable to cast myobundles have been achieved, though cast constructs remain the functional benchmark [140]. Bioprinted murine muscle has also been used to model cancer cachexia *in vitro* [136], supporting future applications in disease modeling and drug testing, including IMAT-muscle crosstalk in metabolic disease where muscle force and insulin sensitivity are key outcomes.

#### 6.4.2 Fat culture: *In vitro* fat models with the potential to study IMAT crosstalk

In parallel to advances in engineered 3D muscle systems, adipocyte cultures have progressed from adherent 2D *in vitro* cultures to increasingly more physiologically relevant 3D cultivation protocols. Traditional monolayer cultures of primary human preadipocytes have provided valuable insight into mechanistic pathways but have not yielded *in vivo*-like, mature, unilocular adipocytes after adipogenic differentiation. Instead, 2D differentiation of preadipocytes mostly results in cells with multilocular lipid droplets, reflecting immature adipocytes [147,148]. Additionally, isolated mature adipocytes from biopsies are considered fragile, their unilocular lipid droplets have been observed to burst, only allowing short-term cultivation. Furthermore, they do not easily adhere to culture dishes due to their buoyancy [149]. A shift towards adipose spheroid cultures based on ASCs addresses some limitations of 2D culture. For one, spheroids are a better representation of *in vivo* structure of white adipose tissue, that is usually present in tightly packed groups, supported by loose connective tissue [150,151]. Most importantly, spheroid cultures support the maturation of adipogenic progenitors, leading to unilocular lipid droplet formation [147,148]. These ASC-derived 3D adipose spheroids present close recapitulations of adipocyte and adipose tissue morphology while exhibiting paracrine activity, making them suitable blueprints for biofabrication of a physiologically relevant 3D IMAT model using actual IMAT-precursors [152], though they do not fully mirror the complexity of adipose tissue with its different cell populations [18] (Figure 5).

#### 6.4.3 Adding fat to the mix: engineering *In vitro* IMAT models

Building on advances in engineered 3D muscle and adipose tissues, the next step toward a physiological IMAT model is their integration. While simple bioassembly of adipocyte spheroids and myobundles could yield a functional construct, IMAT itself remains incompletely characterized and using adipocytes isolated directly from IMAT would undermine the advantages of *in vitro* modeling. A more suitable strategy would mimic *in vivo* co-development through co-differentiation of muscle and adipose precursors. Fibro-adipogenic progenitors (FAPs), proposed native IMAT progenitors [104,153], represent a promising approach. Differentiating human FAPs into adipocytes and incorporating them into engineered muscle could therefore recapitulate the origin of IMAT while enabling the study of maladaptive differentiation. However, co-culture presents technical barriers. Myogenic and adipogenic differentiation requires potentially incompatible media [123,154] while mechanical demands differ, as contractile muscle needs tensioned elastic matrices whereas adipocytes require softer environments [155]. Spatial compartmentalization is also critical to prevent adipocyte overgrowth disrupting myogenic regions.

For such co-culture systems, functional validation is essential. Myobundle contractility can assess the effects of adipocytes on force generation. However, functional readouts within co-culture constructs, especially imaging-based analysis of contractility, could pose a challenge. The physical differences between soft adipose regions and denser myotubes might cause light scattering. An imaging technique that preserves cell functionality and allows 3D insight is needed. High-resolution confocal microscopy with cell-type-specific labeling may enable 3D functional assessment. Dissecting cell-specific metabolic readouts is similarly difficult. For instance, glucose uptake signals cannot easily be assigned to muscle versus adipocytes. Post-culture cell separation via magnetic-activated cell sorting (MACS), fluorescence-activated cell sorting (FACS) or single nucleus RNA sequencing (snRNA seq) may therefore be required prior to analysis. Collectively, *in vitro* IMAT models emerging in the future will provide the first step towards dissecting the cellular and molecular mechanism of IMAT-muscle crosstalk while offering a translational platform for therapeutic screening for diabetes, sarcopenia, cachexia and other diseases causing loss of muscle mass and function.

## 7 Future fat: where do we go from here?

In summary, the accumulating evidence positions IMAT as both a reflection and a likely driver of skeletal muscle health and metabolic dysfunction. It takes on the role of an energy depot that bridges metabolism, inflammation and tissue remodeling. Despite its increasing clinical relevance, IMAT remains sparsely researched compared to other fat depots and is still one of the least understood adipose tissues due to its poor accessibility. If scientists are to translate mechanistic insights into therapeutic strategies, this field of research must move beyond correlative studies and small-scale animal models towards a more human-centered and technology-driven research. The following key questions must be clarified: how IMAT forms, which subtypes and locations are the most harmful and how to best target IMAT. This requires *in vitro* muscle-fat interfaces that mimic IMAT-structure and include muscle-residing cell types. Such *in vitro* models would allow testing of causal hypotheses, e.g. the altered behavior of FAPs in metabolic stress, basic mechanisms of IMAT development and depot-specific effects of IMAT e.g. compared to VAT, while having improved physiological translatability. A comparison of physiologically relevant co-culture systems using IMAT-compared to VAT-derived adipocytes could help uncover unique effects of IMAT on muscle metabolism and function. In parallel, the expansion of multi-omics technologies in combination with artificial intelligence (AI)-assisted image analyses could contribute to expand data on IMAT via MRI/CT-images from clinical studies, leading to the development of digital replications of patient-specific physiology, so-called digital twins. AI models can be trained to distinguish muscle, fat, IMAT and bone in MRI scans to efficiently quantify tissue volume and distribution, enabling large-scale investigation of IMAT across populations and datasets, as published by Anwar et al. [156]. Combining a deep learning model and 3D biofabrication, AI-standardized bioprinted IMAT models in different health conditions, BMI and ages could be achieved which then can be used for fundamental research on development, cellular mechanisms and signaling pathways. Patient-specific models using primary cells from biopsies in these 3D-IMAT constructs would allow for personalized- and precision medicine to be developed further down the line, realizing their digital twins. An individual recreation of metabolic and inflammatory profiles *in vitro* would promote strategies for tailored interventions and drug responses, limiting side effects and elevating treatment effectiveness. Closing back in on general IMAT-models, with this intelligent, bioengineered approach, it may soon be able to predict how specific drugs, exercise regimens or metabolic modulators alter IMAT content and the related muscle function. Ultimately, the establishment of 3D IMAT constructs is a necessary next step, not only in identifying causal mechanisms, but as tools in translational treatments of IMAT-related metabolic dysfunctions. They would serve as a bridge between current mechanistic insight and clinical application, accelerating the development of IMAT-targeted interventions aimed at restoring healthy muscle-fat communication in metabolic disease, aging and cachexia.

## CRediT authorship contribution statement

**Vanessa M. Weik:** Writing – review & editing, Writing – original draft, Data curation, Conceptualization. **Andreas L. Birkenfeld:** Writing – review & editing, Resources. **Andreas Peter:** Writing – review & editing, Resources. **Cora Weigert:** Writing – review & editing, Supervision, Resources, Funding acquisition, Conceptualization. **Simon I. Dreher:** Writing – review & editing, Writing – original draft, Supervision, Methodology, Funding acquisition, Data curation, Conceptualization.

## Ethics declaration

The author(s) declare(s) that the study does not involve humans nor animal subjects.

## Funding

This review was supported in part by grants from the German 10.13039/501100002347Federal Ministry of Education and Research (10.13039/501100002347BMBF) to the German Centre for Diabetes Research (DZD e.V.) under Grant No. 01GI0925. This research is part of the project LSE-021-3D-IMAT, financed by the Baden-Württemberg Stiftung gGmbH within the research program Life Science Engineering received by S.I.D.

## Declaration of competing interest

The authors declare that they have no known competing financial interests or personal relationships that could have appeared to influence the work reported in this paper.

## Data Availability

Data will be made available on request.
